# Detection of Microplastics in Human Bronchoalveolar Lavage Fluid: Preliminary Evidence of Respiratory Exposure to Environmental Contaminants

**DOI:** 10.7759/cureus.97632

**Published:** 2025-11-24

**Authors:** Georgios E Zakynthinos, Ilias E Dimeas, Christos Salmas, Athanasios D Pagonis, Ioannis D Papanikolaou, Evangelos Oikonomou, Konstantinos N Tourlakopoulos, Eleni Karetsi, Zoe Daniil

**Affiliations:** 1 3rd Department of Cardiology, “Sotiria” Chest Diseases Hospital, Medical School, National and Kapodistrian University of Athens, Athens, GRC; 2 Department of Respiratory Medicine, Faculty of Medicine, University of Thessaly, Larissa, GRC; 3 Department of Respiratory Medicine, School of Medicine, University College Dublin, Dublin, IRL; 4 Laboratory of Mineralogy-Geology, Department of Natural Resources and Agricultural Engineering, Agricultural University of Athens, Athens, GRC

**Keywords:** bronchoalveolar lavage, environmental exposure, lung cancer, microplastics, respiratory health

## Abstract

Objectives

Microplastics are increasingly recognised as environmental pollutants of potential concern for human health. Inhalation of airborne particles and their deposition in the respiratory tract may contribute to respiratory disease, yet direct human evidence remains scarce. This study aimed to investigate the presence, morphology, and size of microplastics in bronchoalveolar lavage (BAL) fluid from patients undergoing diagnostic bronchoscopy.

Methods

BAL samples from eight adults were digested with 10% potassium hydroxide, filtered, and examined microscopically under brightfield illumination. Samples were collected in sterilised glass containers to avoid plastic contamination. Microplastics were identified and classified by morphology and measured by Feret’s minimum diameter (dFeretMin). Two blank controls (saline through the bronchoscope and reagent blank) were processed in parallel to assess procedural contamination.

Results

Microplastics were detected in five of eight samples (63%), yielding 22 particles (20 fragments, two fibres). Particle sizes ranged from 5.9 µm to 204.7 µm (mean 44.8 µm), and 80% were below 50 µm, consistent with respirable dimensions. Controls revealed only large fibres (>500 µm) and no fragments, suggesting minimal contamination. The highest burden was observed in a patient with thoracic lymphoma, but given the small sample size, correlations were interpreted descriptively.

Conclusions

This pilot study provides preliminary evidence of microplastics in human BAL, supporting the concept of lower-airway deposition of airborne environmental contaminants. Although limited by visual identification without spectroscopic confirmation, adherence to contamination controls strengthens confidence in the findings and highlights the feasibility of BAL-based human exposure assessment. These results highlight the need to integrate microplastic monitoring into occupational and environmental exposure surveillance.

## Introduction

The presence of microplastics and nanoplastics in the environment has become an increasing concern due to their widespread distribution and potential health impacts. Microplastics, defined as plastic particles less than 5 millimetres in diameter, and nanoplastics, which are even smaller (less than 1 micrometre), originate from the degradation of larger plastic debris and primary sources such as microbeads in personal care products, synthetic textiles, and various industrial processes. As plastics degrade, they break into progressively smaller particles, some of which become airborne, making inhalation exposure a significant concern for human health [1].

Microplastics are ubiquitous across aquatic, terrestrial, and atmospheric environments, even in remote regions [2-7]. Inhalation of airborne particles is particularly concerning because of their potential to deposit in the respiratory tract and cause inflammation or fibrosis [8]. While microplastic contamination has long been associated primarily with aquatic environments, growing evidence suggests that airborne exposure is also a significant and underrecognised route [7]. The World Health Organization has recently highlighted that individuals may inhale up to 3,000 microplastic particles per day [9]. Given links between particulate matter and respiratory harm [10], this airborne burden is a public-health concern amid rising plastic use [11].

Despite the growing awareness of these risks, there remains a considerable gap in understanding the behaviour of microplastics once inhaled and their potential contribution to respiratory disease [12]. Lung cancer, the leading cause of cancer mortality, occurs frequently in never-smokers, suggesting additional environmental contributors such as chronic exposure to airborne particulates, including microplastics [13-15]. Microplastics contain pigments and additives such as phthalates that enhance flexibility and durability but also contribute to their non-biodegradability [16]. These additives may be released during ageing and fragmentation, potentially exerting toxic effects [17].

The potential toxicity of microplastics is linked to several mechanisms, including physical damage to lung tissue, chemical toxicity from adsorbed pollutants, and the induction of oxidative stress and inflammation [11]. These particles can cross biological barriers and accumulate in tissues, raising concerns about their contribution to long-term respiratory effects [18]. However, direct human evidence of inhaled microplastics in the lower airways remains limited [19-21].

This study aims to investigate the presence, morphology, and size of microplastics in bronchoalveolar lavage (BAL) samples collected from patients undergoing diagnostic bronchoscopy. By analysing these samples under strict contamination control, we aim to provide preliminary human evidence of respiratory exposure to environmental microplastics and to evaluate BAL as a feasible matrix for assessing internal exposure and potential implications for pulmonary health.

## Materials and methods

Sample collection and preparation

A total of eight patients who underwent bronchoscopy for diagnostic purposes at the University Hospital of Larissa, Greece, in May 2025, were prospectively included in the study. All patients were permanent residents of the urban area of Thessaly to ensure, as much as possible, uniform environmental exposure to airborne microplastics. Inclusion criteria required diagnostic bronchoscopy with BAL, while patients with oxygen saturation below 94%, haemodynamic instability, or suspected/known contagious diseases were excluded. Additionally, patients with known occupational plastic exposure, prior chemoradiotherapy, or concurrent malignancy were not included.

All participants provided written informed consent after a full explanation. The study complied with the Declaration of Helsinki and received ethics approval from the Human Research Ethics Committee of the University Hospital of Larissa (approval number 22/6th; 29-04-25). Clinical trial registration is not applicable, as this was an observational pilot study and registration was not required. This study focused on methodological feasibility and exposure characterisation rather than clinical outcomes. From each patient, BAL was collected for diagnostic and microplastic analyses, and the final clinical diagnoses of these patients were established through BAL and complementary diagnostic tools.

BAL sampling

BAL sampling was performed according to the standardised technique during bronchoscopy under conscious sedation. A flexible bronchoscope was inserted through the nasal passage into the airways. A 0.9% sodium chloride (NaCl) saline solution was instilled in several stages via the bronchoscope to wash the airways. Briefly, once the fibreoptic bronchoscope was wedged in a bronchus, preferably in the middle lobe or lingula, two successive 50 mL aliquots of filtered and sterilised 0.9% NaCl solution were instilled. Each aliquot was manually aspirated using a 20-mL single-use syringe dedicated to BAL collection. A minimum of 10 mL was required per sample for microplastics analysis. In cases where a lung mass was present, BAL sampling was performed contralateral to the lesion. To prevent secondary contamination, all recovered BAL was collected directly into sterilised glass containers with airtight caps rather than plastic. Immediately, 70% ethanol was added to preserve samples and limit microbial growth. Samples were maintained at 4°C until processing, and then were transferred to glass beakers and topped with filtered deionised water to 50 mL.

Alkaline digestion protocol

Samples underwent alkaline digestion using 10% potassium hydroxide (KOH) solution in a 1:3 ratio (sample: KOH), with each sample reaching a total volume of 200 mL. Digestion proceeded over 96 hours at 60°C on a heating plate [22]. Samples containing blood turned yellow without an exothermic reaction. Digestion was conducted under a laminar flow hood, with all beakers covered in aluminium foil to prevent airborne contamination. Fresh KOH reagent was filtered (0.22 µm) before use and replenished daily to account for evaporation and maintain effective digestion conditions. All personnel wore cotton laboratory coats and nitrile gloves to minimise synthetic fibre shedding.

Filtration and microscopy

Digested samples were vacuum-filtered using Macherey-Nagel cellulose nitrate membrane filters (0.47 μm pore size; Macherey-Nagel™ Porafil™ CM Membrane Filters 65300045047 (Düren, Germany)). Filters were dried for 24 hours in sterile glass Petri dishes inside a fume hood at room temperature (~23°C). Laboratory protocols to minimise contamination included working in sterilised environments, using ethanol (95%) for surface cleaning, and wearing cotton lab coats, nitrile gloves, face masks, and protective eyewear. Only glassware pre-rinsed with filtered deionised water and inspected under light for debris was used.

Controls

Two negative controls (blanks) were processed in parallel: one containing 4 litres of deionised water and another undergoing the full digestion process without biological material (deionised water + 10% KOH). These controls served to quantify potential background contamination from reagents or laboratory handling. In addition, a procedural blank consisting of saline passed through the bronchoscope before patient sampling was included to monitor potential instrument-related contamination.

Microparticle identification and quantification in the microscope

Microparticles were quantified using a Leica IVESTA 3 stereomicroscope (Leica Microsystems, Wetzlar, Germany) with a Flexacam C5 camera at a maximum magnification of 55×. Particles suspected to be microplastics based on their morphology and colour were further analysed using a Leica DM750P transmitted light (brightfield) microscope (Leica Microsystems) with a Leica ICC50 W camera (Leica Microsystems) at 40×, 100×, and 400× magnifications. Image processing and measurement were performed using Leica LAS X software (Leica Microsystems). All filter areas were examined, and two dimensions were measured per particle, and Feret’s minimum diameter (dFeretMin) was used for statistical analysis, consistent with previous studies. Spectroscopic confirmation, such as Raman or micro-Fourier transform infrared spectroscopy (µ-FTIR), was not performed due to sample size constraints; however, morphology-based identification under controlled laboratory conditions remains an accepted preliminary approach for exploratory exposure studies.

## Results

A total of eight patients were included in the study, comprising five males and three females. The mean age of the cohort was 56.30 years, with ages ranging from 22 to 70 years. Four of the patients were active or former smokers, with smoking histories ranging from 40 to 60 pack-years, while the remaining four were lifelong non-smokers. All patients resided in the urban area of Larissa, ensuring comparable environmental exposure. No participant had known occupational exposure to plastics or industrial environments with documented airborne particulate emissions, nor had they received recent chemoradiotherapy or had any concurrent malignancies aside from their primary diagnosis (Table 1).

**Table 1:** Patient demographics and smoking history by diagnosis *In the parentheses beside the smoking habit, the p/y of smoking is indicated; p/y represent the number of packs of cigarettes smoked per day multiplied by the number of years the person has smoked. p/y: pack-years

Sample	Age (years)	Smoking habits	Final diagnosis
A	42	No	Carcinoid tumor
B	70	Yes (50 p/y)*	Lung adenocarcinoma
C	22	No	Control (no lung involvement)
D	67	Yes (60 p/y)	Lung adenocarcinoma
E	68	No	Pulmonary aspergillosis
F	50	Yes (40 p/y)	Non-Hodgkin lymphoma with thoracic involvement
G	64	Yes (50 p/y)	Non other specified lung cancer
H	68	No	Control - Endometrial carcinoma (no lung involvement)

**Figure 1:**
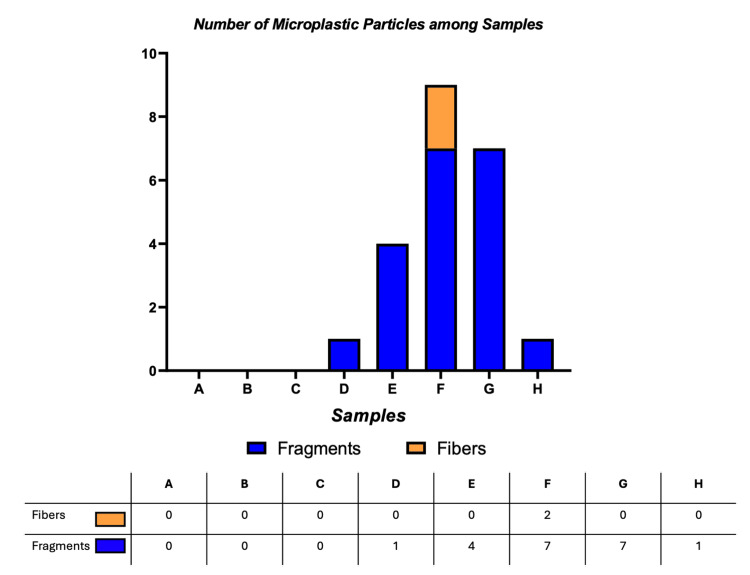
Bars showing the number of fragments and fibers in each sample

All bronchoscopic procedures were performed for diagnostic purposes, and BAL fluid was collected during the same session. Microplastics were identified in five out of eight patient samples. A total of 22 particles were detected: 20 fragments (91%) and two fibres (9%). All microplastic-like particles were visually distinct from mineral dust, biological debris, or organic fibres, based on surface texture, colour uniformity, and lack of cellular morphology. No microplastics were found in BAL samples from Samples A, B, and C (Figure 1, Tables 2, 3).

**Table 2:** The total number of fragments observed in the samples

Sample	Type	Color	Dimension 1 (μm)	Dimension 2 (μm)	dFeretMin (μm)
D	Fragment	Blue	138.46	33.22	33.22
E	Fragment	Blue	37.81	43.22	37.81
E	Fragment	Blue	79.91	62.43	62.43
E	Fragment	Red	30.00	32.00	30.00
E	Fragment	Yellow	38.44	35.77	35.77
F	Fragment	Blue	80.78	82.33	80.78
F	Fragment	Blue	10.65	15.23	10.65
F	Fragment	Blue	37.89	5.90	5.90
F	Fragment	Blue	59.78	12.80	12.80
F	Fragment	Blue	34.11	39.11	34.11
F	Fragment	Transparent	47.37	47.01	47.01
F	Fragment	Transparent	232.14	204.73	204.73
G	Fragment	Blue	68.29	26.72	26.72
G	Fragment	Blue	47.01	32.34	32.34
G	Fragment	Blue	42.05	15.69	15.69
G	Fragment	Blue	10.93	7.10	7.10
G	Fragment	Blue	77.99	47.69	47.69
G	Fragment	White	120.00	120.00	120.00
G	Fragment	Blue	25.00	21.00	21.00
H	Fragment	Blue	35.00	31.00	31.00
	Total Fragments	Total Colors	Mean D1 ± SD (μm)	MeanD2 ± SD (μm)	Mean dFeretMin ± SD (μm)
	20	Blue: 15 Red: 1 Yellow: 1 Transparent: 2 White: 1	62.68 ± 51.72	45.76 ± 46.04	44.84 ± 46.26

**Table 3:** The total number of fibers observed in the samples

Sample	Type	Color	Dimension 1: Length (μm)	Dimension 2: Width (μm)
F	Fiber	Blue	151.00	18.00
F	Fiber	Blue	129.00	18.00
	Total Fibers	Total Colors	Mean Length ± SD (μm)	Mean Width ± SD (μm)
	2	Blue: 2	140.00 ± 15.56	18.00 ± 0.00

**Figure 2:**
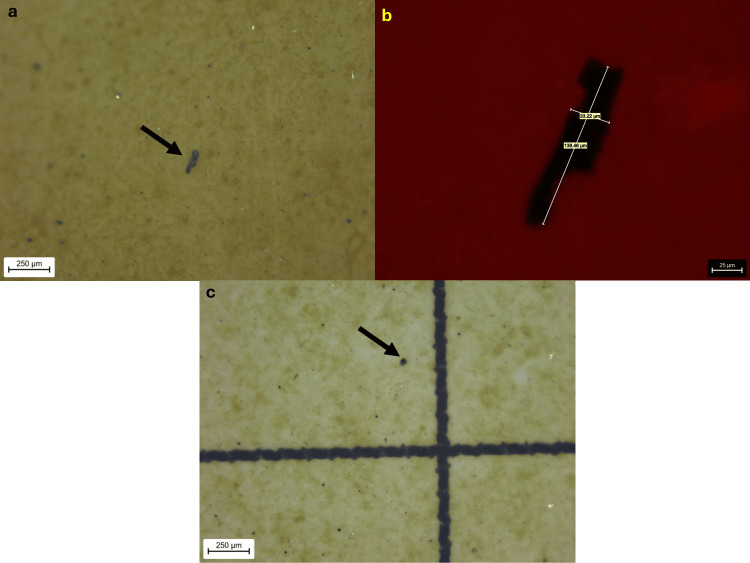
Blue fragment of Sample D and blue fragment of Sample H (a) Blue fragment of Sample D at 55x stereomicroscopic magnification; (b) The same fragment at 400x microscopic magnification; (c) Blue fragment of Sample H

**Figure 3:**
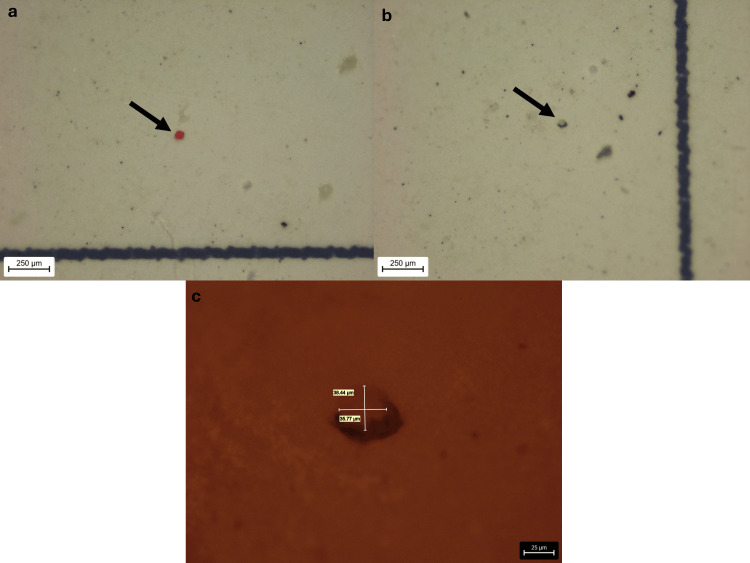
Red fragment of Sample E (a) Red fragment of Sample E at 55x stereomicroscopic magnification; (b, c) Yellow fragment of Sample E at 55x stereomicroscopic magnification and at 400x microscopic magnification.

**Figure 4:**
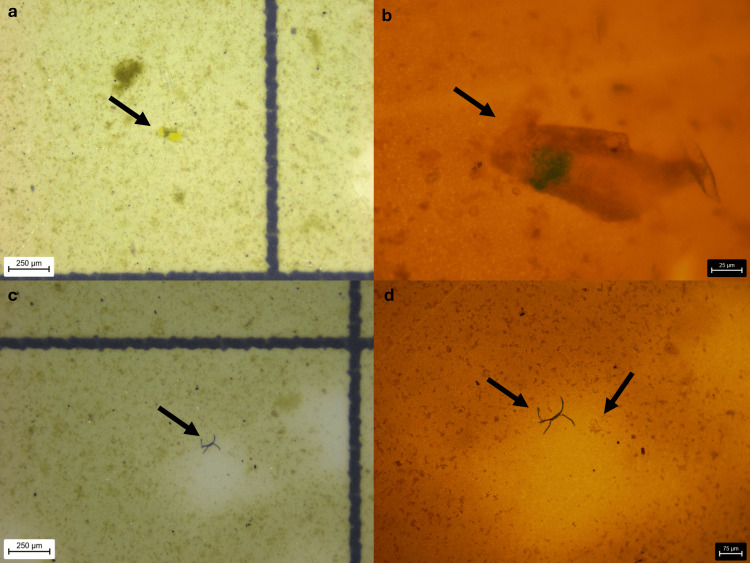
Blue fragment of Sample F (a, b) Blue fragment of Sample F at 55x stereomicroscopic magnification and 400x microscopic magnification; (c, d) Blue fibres of Sample F at 55x stereomicroscopic magnification and 400x microscopic magnification. A transparent fragment is also visible under the microscope, which is difficult to observe stereoscopically.

**Figure 5:**
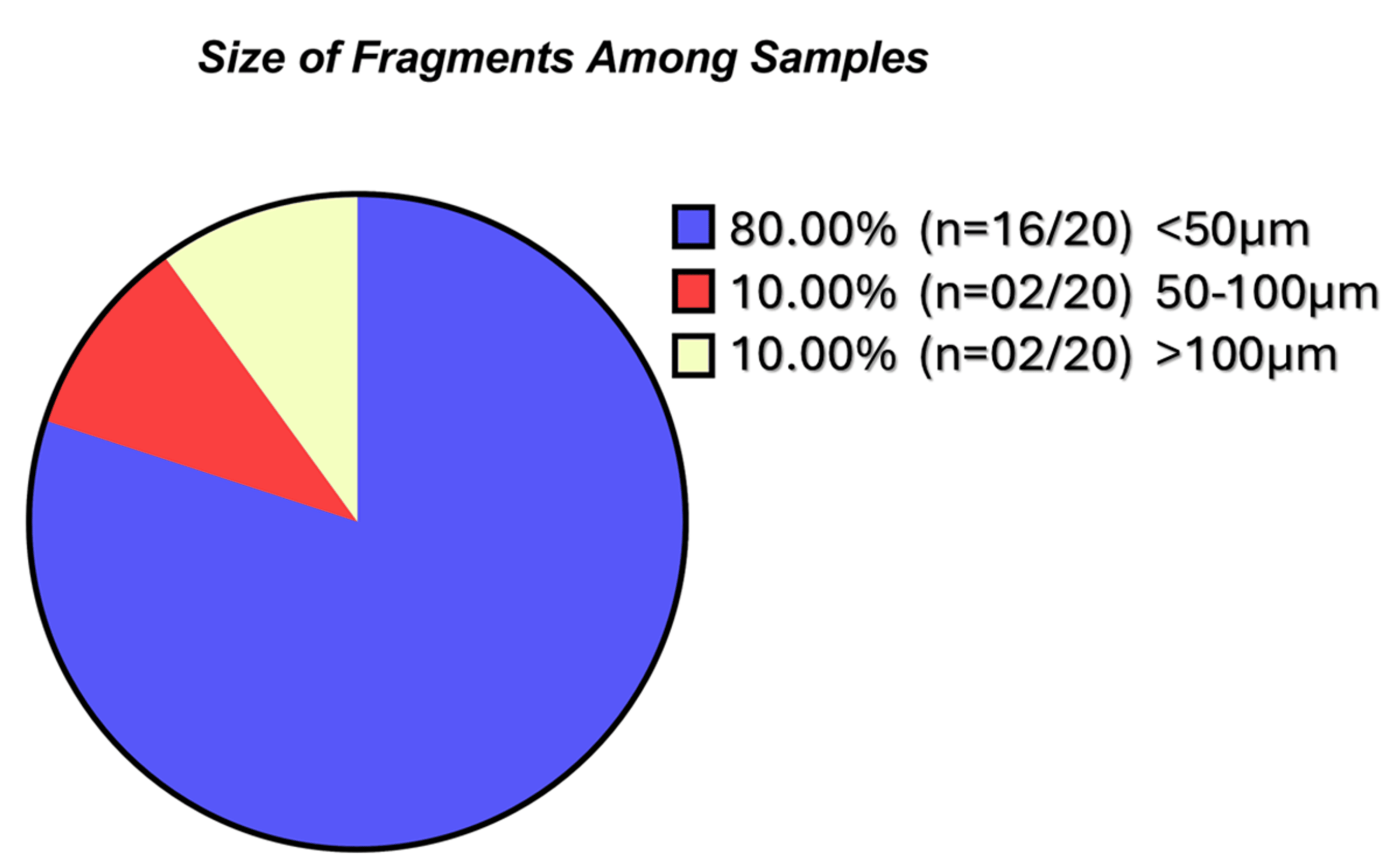
Pie chart indicating the distribution of identified fragments size

The detected microplastic fragments exhibited a wide range of sizes, with FeretMin diameters ranging from 5.90 μm to 204.73 μm and a mean value of 44.84 μm. The smallest FeretMin dimension (5.9 μm) was observed in a particle from Sample F, which also exhibited the smallest secondary dimension (width) of 5.9 μm and included an additional fragment with a minimal dimension of 10.65 μm. Overall, 80% of the identified fragments (16 out of 20) were smaller than 50 μm, and 31% (five out of 16) were under 20 μm, highlighting the predominance of subvisible-sized particles (Figures 2-5).

**Figure 6:**
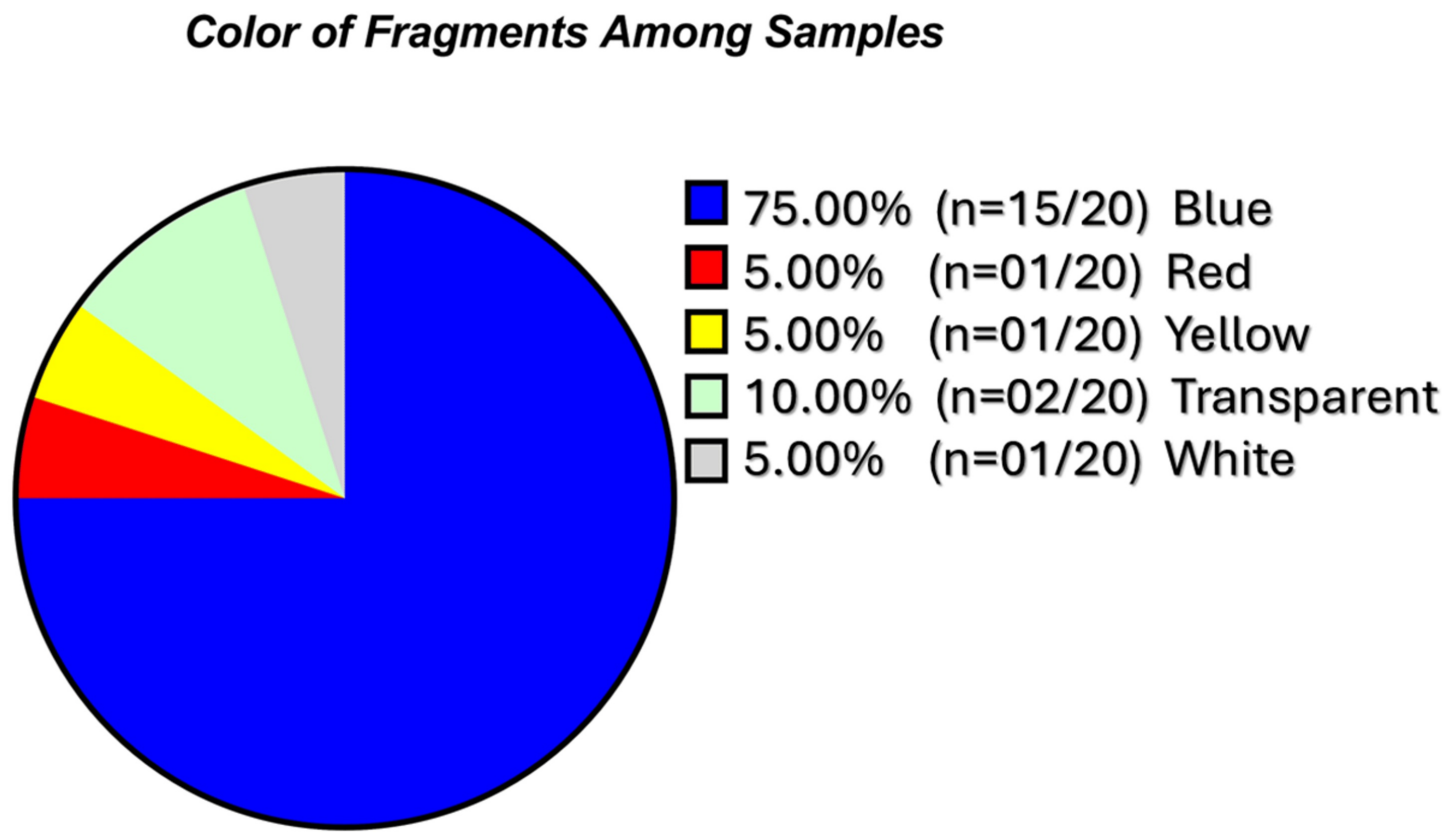
Pie chart indicating the color of the identified fragments

Such particle dimensions fall within the respirable range capable of deposition in terminal bronchioles and alveoli, supporting the plausibility of lower-airway exposure. Fibrous microplastics were exclusively identified in Sample F, with measured lengths of 129 μm and 151 μm and a consistent width of 18 μm. These findings suggest the presence of both fragmented and fibrous microplastics, consistent with airborne environmental sources. Seventy-five percent of fragments and both fibres were blue, consistent with synthetic polymer dyes (Figure 6).

The highest microplastic burden was observed in Sample F, where a total of nine particles were detected: seven fragments and two fibres. This sample alone accounted for 35% of all fragments identified in the study and included 100% of the fibres. In contrast, Samples D, E, G, and H demonstrated lower microplastic loads, each containing between one and four fragments. This heterogeneity may reflect inter-individual differences in recent exposure, airway clearance efficiency, or disease-related retention rather than laboratory variability.

Control samples (blanks) revealed the presence of six fibres (>500 μm) in the deionised water control and 3 fibres (>500 μm) in the procedural blank. However, no fragments were identified in either control. The absence of small fragments in all blanks, combined with strict use of glassware and filtered reagents, suggests that cross-contamination from laboratory handling was minimal. The significantly smaller size of the two fibres found in the patient samples, their consistency with the literature in terms of human tissue detection, and the absence of fragments in the controls suggest that contamination during laboratory processing is unlikely to account for the findings.

The presence of microplastics varied across the different patient diagnoses, as shown in Table 4.

**Table 4:** Microplastic detection by patient and diagnosis

Sample	Final diagnosis	Total microplastics detected	Fragments (n)	Fibres (n)
A	Carcinoid tumor	0	0	0
B	Lung adenocarcinoma	0	0	0
C	Control (no lung involvement)	0	0	0
D	Lung adenocarcinoma	1	1	0
E	Pulmonary aspergillosis	4	4	0
F	Non-Hodgkin lymphoma with thoracic involvement	9	7	2
G	Non-other specified lung cancer	7	7	0
H	Endometrial carcinoma (no lung involvement)	1	1	0

The highest number of particles, including both fragments and fibres, was observed in Sample F, which had a diagnosis of non-Hodgkin lymphoma (NHL) with thoracic involvement. This sample contained nine microplastic particles (seven fragments and two fibres), representing 35% of all fragments detected in the study. Samples from patients diagnosed with NHL (Samples F and G), which both involved pulmonary involvement, also showed microplastics, though the numbers were more variable. Sample G contained four fragments, while Sample F had the highest particle burden with seven fragments and two fibres. In contrast, Samples A (carcinoid tumour) and C (control, no lung involvement) showed no microplastic contamination, underscoring the non-uniform distribution of detected microplastics across diagnostic categories. Sample H, diagnosed with endometrial carcinoma, showed one fragment (35 μm × 31 μm), indicating that microplastic presence could be less prevalent in this cancer type, although the single detected particle does not completely rule out the possibility of exposure. No causal interpretation was attempted, given the pilot design and limited sample size.

A slight trend in the number of microplastic particles correlating with age was observed, with older patients (aged 60 and above) tending to have a higher presence of microplastics in their BAL samples. This observation may reflect cumulative inhalation exposure and particle retention over time, although conclusions are limited by sample size.

A secondary analysis examined the relationship between smoking habit (measured in pack-years) and microplastic presence. Sample F, a smoker with 40 pack-years, exhibited the highest microplastic burden. While the correlation between smoking and microplastic presence was not statistically significant, smoking history may serve as a surrogate for prolonged airway exposure to combustion-derived and ambient particulates, potentially influencing microplastic deposition or clearance.

## Discussion

Our findings provide evidence supporting the presence of microplastics in the lower respiratory tract of patients undergoing diagnostic bronchoscopy. Specifically, microplastic fragments were identified in the BAL samples of five out of eight participants, totalling 22 particles, predominantly fragments and a smaller proportion of fibres. These findings are consistent with the existing literature, as other studies have also detected microplastics in BAL samples [9], as well as in lung tissue. In a study using post-mortem material from 20 non-smokers, 31 microplastics were identified: similarly to our results, 87.5% were particles (all fragments) and 12.5% were fibres [23]. This was confirmed in lung samples obtained from living non-smokers via video-assisted thoracoscopic surgery [24]. Microplastics have also been detected and quantified in the nasal cavity, predominantly fragments, suggesting environmental inhalation as a primary route of entry into the respiratory tract [25]. Larger microplastics tend to be located in the upper airways, with particle size generally decreasing toward the lower respiratory tract, as demonstrated in multiple studies [25]. Similar to our findings, almost all published studies on BAL samples report a predominance of fragments, with fragment percentages consistently above 70% [26-28]. However, Momeni et al., who analysed microplastics in sputum, BAL, and pleural fluid, reported a fibre prevalence of 70%. Notably, they also found that, similar to our observations, the proportion of fibre- or line-shaped particles decreased from the upper to the lower respiratory tract, while the proportion of fragments or spherical particles increased [29].

Eighty percent of the identified fragments in our study were under 50 μm in FeretMin diameter, aligning with previous human studies [26-28]. Particle size is a critical determinant of airway deposition: particles < 2.5 μm can reach the alveoli, 2.5-100 μm deposit in larger airways, and submicron particles may translocate systemically [30,31]. The morphological heterogeneity, ranging from irregular fragments to elongated fibres, and the predominance of blue particles are consistent with environmental and autopsy-based observations [28,29], although visual detection bias may contribute to over-representation of coloured particles due to stronger optical contrast under light microscopy. Sample F, which exhibited the highest microplastic burden and included the only two fibrous particles, may reflect increased exposure, impaired clearance, or both. The variability in microplastic load between patients with similar environmental backgrounds highlights potential differences in susceptibility, airway clearance, or disease-related retention. More details about the characteristics of microplastics among studies are shown in Table 5.

**Table 5:** Detection of microplastics in human respiratory samples: a summary of key studies

Study	Country	Population	Specimen	Number of microplastics (MPs)	Type of MPs	Size	Color
Bronchoalveolar lavage (BAL)							
Qiu et al., 2023 [26]	China	24 samples from non-smokers; 88.9% had lung cancer	18 BAL and six control	1634 synthetic polymer particles and fibres, 16 particles in control	4.2% fibres, 95.8% irregular fibres; polyethene 9.5-100% of MPs in 14 BAL samples (77.8%). Polyethene terephthalate accounted for 0.4%−92% in 16 samples (88.9%)	20-80 μm; median diameter of 34.0 μm	Not mentioned
Uoginte et al., 2023 [27]	Lithuania	10 randomly selected patients	BAL	All patients had MPs	Fragments: 84.42%, fibres: 15.65%	Width of particles: 20 μm - 283 μm (average of 49 μm), length of particles: 35 μm - 1020 μm (average of 203 μm)	Not mentioned
Ozgen Alpaydin et al., 2024 [28]	Turkey	Patients who underwent fiberoptic bronchoscopy with BAL, the Interstitial lung disease (ILD) group (n= 18), and the control group (n= 7)	BAL	10 of 18 patients (55%) in the ILD-suspected, and two of seven (29%) control group had MPs	Particle: 80.0% The dominant polymer types in BAL samples were polyamide, polyester, polyvinyl chloride, and polyurethanes, with a ratio of 20.0%	4.19-792 μm	Grey/white: 46.7%, black: 33.3%
Monemi et al., 2025 [29]	Iran	34 patients with lung disease	BAL (+Sputum, +Pleural fluid)	All patients had MPs	Fibre/line (about 70%) and fragment/spherical (nearly 30%) polyester, approximately 60%-70% and 8.2% polyamides	Largest 1135 μm, smallest 11 μm <100 μm, 50% of samples	White-transparent (about 52%), black-grey-brown (21%), blue-green (15%), red-pink (11%), and yellow-orange (about 1%)
Baeza-Martínez et al., 2022 [32]	Spain	44 adult patients, undergoing a bronchoscopy, lung mass (32%), hemoptysis (27%), main diagnosis pulmonary neoplasia (50%)	BAL	14 participants (31.82%) did not have MPs, 12 (27.27%) had only one, and 18 patients (40.91%) had two or more MPs. Average concentration of 9.75 ± 2.49 items/100 mL	Microfibers: 97.06%, particulate MPs: 5.88%	1.73 ± 0.15 mm, with the longest dimension (9.96 mm)	White (51.04%), blue (23.96%), red (7.29%), black (6.25%), and brown (6.25%)
Lu et al., 2023 [33]	China	32 recruited subjects - 17 smokers and 15 nonsmokers	BAL	Smokers: 3.48 to 81.37 particles/g, Non-smokers: 2.04 to 57.38 particles/g	Smokers mean (SD): polyurethane 11.34 (16.38), polyethylene 8.06 (19.98), silicone 1.15 (1.17) particles/g; Non-smokers: polyurethane 4.43 (7.69), poly(ethylene terephthalate) 3.34 (10.14), polyethylene 2.22 (3.13)	20 and 100 μm in diameter	Not mentioned
Lung tissue							
Amato-Lourenço et al., 2021 [23]	Brazil	20 non-smoking adult individuals who underwent routine coroner autopsy for the verification of the cause of death	Pulmonary tissue samples (parenchymal tissue from the distal and proximal regions of the left lung)	13/20 had MPs, 31 synthetic polymer particles and fibres	Particles (all fragments): 87.5%, fibres: 12.5% polymer: 35.1%, polyethylene: 24.3%, cotton: 16.2%	Mean particle size: 3.92 (±0.67) μm, 1.60 - 5.56 μm, mean fibre length: 11.23 (±1.96) μm, 8.12 -16.80 μm	Not mentioned
Wang et al., 2023 [24]	China	12 nonsmoking patients who were finally diagnosed with lung cancer	Video-assisted thoracoscopic surgery (VATS) lobectomy	MPs were detected in all samples except for the lung tissue sample nine and control sample one; MPs in the lung tissue group were significantly higher than those in the control group	108 MPs, including 19 fibres (17.59%), polyethylene terephthalate 21.30%, polystyrene 8.33%, polyvinylchloride 6.48%	20 and 100 μm in diameter, with a median value of 41.36	Not mentioned
Zhao et al., 2024 [34]	China	61 patients aged between 18 and 75 years with one malignant tumour (lung cancer, cervical cancer, etc); 10 patients with lung cancer	Tumour samples - Surgery	26/61 samples had MPs. 8/10 samples of tissues with lung cancer had MPs	Polystyrene emerged as the predominant MP polymer type: 20 samples (59.56 ± 89.15 ng/g). Polyvinyl chloride and polyethylene were identified in 17 and 11 of the analysed samples	Not mentioned	Not mentioned
Jenner et al., 2022 [35]	United Kingdom	13 patients following surgical resection for cancer or lung volume reduction surgery	Peripheral human lung tissue collected from the upper, middle (left lingula), or lower lobe	11/13 tissues had MPs (number: 39 MPs)	Fibre: 19, 49%, fragment: 17, 43%, film: 3, 8% PP: 9, 23%, PET: 7, 18% - the most abundant	Mean particle length: 223.10 ± 436.16 μm (range 12-2475 μm) Mean particle width: 22.21 ± 20.32 μm (range 4-88 μm)	Not mentioned

We acknowledge that the absence of spectroscopic confirmation (e.g., μ-FTIR, Raman) represents a methodological limitation, as misclassification or confusion with natural fibres cannot be fully excluded. However, contamination controls processed in parallel did not yield microplastic-like fragments, supporting the robustness of our findings. The literature also indicates that laboratory contamination most commonly involves fibrous particles rather than irregular fragments [36]; thus, the predominance of fragments in our samples argues against artefactual contamination. Nevertheless, future work using chemical confirmation techniques will be essential to validate these preliminary observations.

Microplastics were detected in the BAL of five out of eight patients, though the absolute numbers were low. Notably, one patient with thoracic involvement of NHL exhibited the highest burden (nine particles, including the only two fibres), while others, including controls, showed minimal or absent microplastic loads. These findings are descriptive and hypothesis-generating rather than inferential.

Several studies have reported microplastics in a range of malignancies and chronic lung diseases [1,9,21]. In tumour tissue, detection rates vary widely, with lung cancer consistently showing the highest prevalence compared with other cancer types [34]. Microplastics have also been identified in conditions such as asthma, chronic obstructive pulmonary disease, fibrosis, and pulmonary nodules, suggesting that diseased or inflamed tissue may facilitate retention [30]. Collectively, microplastics occur across disease states, although causality remains unproven.

To improve clarity, we provide a summary in Table 4, which presents our patient-level results by diagnosis. This allows direct visualisation of inter-individual variability and situates our preliminary findings in the broader context of microplastic detection in human disease. The observed trend linking microplastic burden with age, particularly among patients aged 60 and above, is consistent with the cumulative nature of environmental exposure. Given the persistent and ubiquitous presence of microplastics in air, water, and food, older individuals may have a greater lifetime burden, resulting in detectable quantities in the lungs. This association was observed despite the relatively uniform environmental background of the study population, suggesting that age-related changes such as reduced mucociliary clearance or chronic inflammation may contribute to retention. Some reports also found higher microplastic concentrations in older individuals (4.87 ± 0.30 vs 2.76 ± 0.12 items/100 mL BAL; p < 0.05), whereas others showed no association [29,32]. Given our small sample, this trend should be considered exploratory and warrants confirmation in larger cohorts.

In terms of smoking history, we observed a trend toward higher microplastic loads in individuals with longer smoking exposure. Sample F, a smoker with a 40 pack-year history, not only had the highest overall microplastic count but also was the only case with detectable fibres. While this association was not statistically significant, it supports the hypothesis that chronic inhalational exposures, such as tobacco smoke, may compromise mucosal barriers or impair clearance mechanisms, thereby enhancing microplastic accumulation. This observation is particularly relevant given the shared routes of exposure between tobacco-related and airborne pollutants. Indeed, the literature also supports the association between microplastics and smoking (whether active or passive) as well as occupational exposure (p = 0.045), smoking (p = 0.029), since smokers have higher microplastic loads in BAL and tissues [29,32]. Additionally, microplastics have been identified in cigarette smoke [33], and the percentage of microplastics increases in individuals who are simply exposed to it (exposure to cigarette smoke, p = 0.007) [29]. Cigarette tubes may also release microplastics during smoking since they have filters and adhesives. Cigarette filters are often made from petro-based fibres (nylon and polyester). These findings highlight the relevance of co-exposure assessment in environmental health studies and support the inclusion of smoking as an exposure modifier in future analyses.

Contamination control was rigorously implemented throughout all analytical stages. While a small number of fibres (>500 μm) were observed in both the deionised water and procedural blanks, no microplastic fragments, the predominant particle type identified in patient samples, were detected in the control runs. Furthermore, the fibres observed in controls were significantly larger than those detected in clinical samples, and none of the distinctive smaller fragments were present. These findings, combined with adherence to strict laboratory protocols and cleanroom conditions, suggest that the presence of microplastics in BAL samples reflects true biological occurrence rather than laboratory artifact/contamination. Indeed, a study from Greece showed that in the environment, in spaces where we circulate, and in materials to which we are exposed, fibres are primarily present and not fragments that we found in BAL [37]. This contrast strengthens the inference that the detected fragments likely represent inhaled and retained particles rather than artefactual contamination.

This study has several limitations. First, the sample size was small, which limits statistical power and generalizability. Second, the absence of longitudinal follow-up precludes assessment of cumulative or chronic effects of microplastic exposure. Third, particle identification relied on visual inspection without spectroscopic confirmation, which introduces uncertainty, particularly for fibre-like structures. However, strict contamination controls were applied and yielded no microplastic-like fragments; furthermore, the predominance of fragments over fibres reduces the likelihood that findings are attributable to laboratory contamination, as reported contamination events in the literature typically involve fibrous particles. Fourth, although all participants were recruited from the same urban region and hospital, unmeasured environmental, occupational, or lifestyle-related exposures cannot be fully excluded. Finally, the cross-sectional design prevents inference of temporal or causal relationships between microplastic exposure and disease. Future studies using spectroscopic confirmation, standardised protocols, and larger longitudinal cohorts will be essential to validate and extend these findings.

Overall, our results suggest that microplastics may be present in the lungs of patients with specific oncological conditions, particularly those involving the thoracic region. This study raises important questions about the potential role of microplastics in disease progression and lung health. While the study's small sample size and observational design limit the ability to establish definitive causal relationships, the findings contribute to the growing body of evidence suggesting that microplastics are ubiquitous in the human body and may play a role in pulmonary pathology. Future studies integrating spectroscopic confirmation, quantitative exposure metrics, and mechanistic models are warranted to clarify exposure-response relationships and to inform risk assessment in occupational and environmental health.

## Conclusions

This study demonstrates the presence of microplastic particles in BAL samples from patients with various oncological diagnoses. These findings provide preliminary human evidence of lower-airway exposure to environmental microplastics and raise hypotheses for future evaluation regarding disease-related retention. While age and smoking history appeared to influence microplastic presence, these observations should be interpreted cautiously, given the small sample size and descriptive study design. This exploratory work supports the methodological feasibility of BAL as a matrix for internal exposure assessment and emphasises the need for larger, spectroscopically confirmed studies to validate and expand upon these findings. A better understanding of the pathways and biological behaviour of inhaled microplastics will be essential to determine their potential relevance for environmental and occupational health.
